# Drinking Water Quality Surveillance Information System (SISAGUA): evaluation of data completeness on water supply coverage, Brazil, 2014-2020

**DOI:** 10.1590/S2237-96222022000300003

**Published:** 2022-09-05

**Authors:** Renan Neves da Mata, Aristeu de Oliveira, Walter Massa Ramalho

**Affiliations:** 1 Universidade de Brasília, Programa de Pós-Graduação em Saúde Coletiva, Brasília, DF, Brazil; 2 Ministério da Saúde, Departamento de Saúde Ambiental e Saúde do Trabalhador, Brasília, DF, Brazil

**Keywords:** Drinking Water, Health Information Systems, Public Health Surveillance, Environmental Health, Descriptive Epidemiology

## Abstract

**Objective:**

To evaluate the completeness of dataset of the Drinking Water Quality Surveillance Information System (SISAGUA) regarding information on the coverage of water supply for human consumption in Brazil.

**Methods:**

This was a descriptive study on data between 2014 and 2020. A relative frequency distribution of 35 variables was calculated. Completeness was categorized as excellent (≥ 95%), good (90% to 94%), regular (70% to 89%), poor (50% to 69%) and very poor (≤ 49%).

**Results:**

In the period, there were 861,250 records of forms of water supply. With regard to data completeness, SISAGUA obtained an excellent classification for 25 variables, good for two, regular for three, poor for one and very poor for four variables.

**Conclusion:**

The system showed excellent data completeness for most of the variables. This type of study contributes to the continuous improvement of SISAGUA and enables the identification of inconsistencies and weaknesses.

**Study contributions**

**Main results**

The Drinking Water Quality Surveillance Information System (SISAGUA) showed excellent data completeness for most of the variables: excellent (25), good (2), regular (3), poor (1) and very poor (4).

**Implications for services**

The evaluation of completeness of SISAGUA makes it possible to check the quality of the system data, as well as to identify areas to be improved, supporting its use in the surveillance of the quality of water for human consumption.

**Perspectives**

The incompleteness of some SISAGUA variables points to the need for continuous improvement of the system and investment in the user training process and raising awareness of the importance of filling out the fields properly.

## Introduction

The Ministry of Health of Brazil monitors the quality of water consumed by the population through the National Drinking Water Quality Surveillance Program (VIGIAGUA).^1^ Drinking Water Surveillance Information System (SISAGUA) is one of the instruments of the VIGIAGUA. It is made available to health departments and water supply service providers in order for them to enter their monitoring data.^2^

It is possible to register the three types of water supply on SISAGUA: Water Supply System (SAA) - a system aimed at drinking water production and collective supply, by means of a distribution network; Alternative Collective Solution (SAC), mode of collective supplies intended to provide drinking water, with or without pipeline and without distribution network; and Alternative Individual Solution (SAI), mode of water supply for human consumption serving single family residences.^3^

In 2015, about three out of ten people (2.1 billion individuals, or 29% of the world's population) did not have access to a safely managed drinking water service, and 844 million still lacked a basic potable water service.^4^ ^,^ ^5^

SISAGUA data provide information at the national level on the coverage of water supply for human consumption in the country. However, studies evaluating the quality of these data are still scarce. The objective of this study was to evaluate the completeness of SISAGUA records between 2014 and 2020.

## Methods

This was a descriptive study on data completeness in SISAGUA regarding the coverage of water supply system in Brazil. This dataset has information on the number of households supplied by alternative water supply systems and solutions.

The period analyzed was between 2014 and 2020, and its data are made available on the Brazilian Open Data Portal.^6^ They were consulted on May 19, 2021. On-screen analysis followed the criteria of the Centers for Disease Control and Prevention (CDC), according to which the completeness of a health information system consists of the degree of completion of each field analyzed and it is measured by the proportion between filled fields and unfilled fields.^7^

Microsoft Excel 365 was used for data processing. The completeness of 35 variables was calculated as the proportion of filled fields in relation to the total of records for each year, subsequently, the average of the results was calculated to represent the analyzed period.

SISAGUA’s rules were not taken into consideration for the calculation in the initial completeness. The ordering of variables followed a descending order, according to this result. In the final completeness, rules of both the data dictionary^6^ and SISAGUA’s manuals were taken into account.^8^ ^,^ ^9^ This has enabled a more reliable analysis, since the necessary filters were applied in order to verify the relevance or not of filling in the fields. The analysis of the variables was performed individually, however, the grouped results are due to the rules and structure of the system.

The final incompleteness corresponded to the subtraction of 100% by the value found in the average percentage of final completeness. Completeness was classified as excellent (≥ 95%), good (90% to 94%), regular (70% to 89%), poor (50% to 69%) and very poor (≤ 49%).^10^

## Results

Between 2014 and 2020, 861,250 records were identified regarding the forms of water supply in Brazil, of which 96,723 were records for SAA, 354,091 for SAC and 410,436 for SAI.

**Table 1:** Annual percentage of final completeness of records of forms of water supply, Drinking Water Quality Surveillance Information System (SISAGUA), Brazil, 2014-2020

Variable	Percentage of completeness						
	2014	2015	2016	2017	2018	2019	2020
Geographical region^a^	100.0	100.0	100.0	100.0	100.0	100.0	100.0
Federative Unit	100.0	100.0	100.0	100.0	100.0	100.0	100.0
Regional Health Care^a^	100.0	100.0	100.0	100.0	100.0	100.0	100.0
Municipality^a^	100.0	100.0	100.0	100.0	100.0	100.0	100.0
IBGE Code^a,b^	100.0	100.0	100.0	100.0	100.0	100.0	100.0
Type of form of supply^a^	100.0	100.0	100.0	100.0	100.0	100.0	100.0
Form of water supply code^a^	100.0	100.0	100.0	100.0	100.0	100.0	100.0
Name of the form of water supply^a^	100.0	100.0	100.0	100.0	100.0	100.0	100.0
Reference year^a^	100.0	100.0	100.0	100.0	100.0	100.0	100.0
Date of registration^a^	100.0	100.0	100.0	100.0	100.0	100.0	100.0
Date of completion^a^	100.0	100.0	100.0	100.0	100.0	100.0	100.0
Surface water collection^a^	100.0	100.0	100.0	100.0	100.0	100.0	100.0
Underground water collection^a^	100.0	100.0	100.0	100.0	100.0	100.0	100.0
Inhabitant/household ratio^a^	100.0	100.0	100.0	100.0	100.0	100.0	100.0
Number of residential economies (permanent households)^a^	99.9	100.0	99.9	99.9	100.0	100.0	100.0
Filtration^c^	94.2	94.0	94.8	95.7	95.3	96.0	96.3
Disinfection^c^	93.5	93.1	94.5	95.3	94.9	95.6	96.0
Cistern^d^	84.7	87.7	88.3	90.1	89.4	89.5	89.4
Rainwater harvesting^d^	84.7	87.7	88.3	90.1	89.4	89.5	89.4
Water tank^d^	44.9	47.7	46.4	52.4	46.5	47.1	47.0
No reservoir^d^	44.9	47.7	46.4	52.4	46.5	47.1	47.0
SAA/SAC provide water to the population^c,e,f^	44.9	47.7	46.4	52.4	46.5	47.1	47.0
Water tank truck^d^	39.9	40.0	41.8	37.7	42.9	42.4	42.4
Fountain^d^	39.9	40.0	41.8	37.7	42.9	42.4	42.4
Spring^d^	39.9	40.0	41.8	37.7	42.9	42.4	42.4
Pipeline^d^	39.9	40.0	41.8	37.7	42.9	42.4	42.4
SAAc,^e^ provides water to the population	39.9	40.0	41.8	37.7	42.9	42.4	42.4
Number of residential economies (occasional use)^d^	33.9	33.4	38.8	38.2	44.6	43.2	43.7
Institution type^d^	40.2	38.0	38.9	35.3	39.7	39.3	39.6
Institution name^d^	40.2	38.0	38.9	35.3	39.7	39.3	39.6
CNPJ of the institution^d^	40.2	38.0	38.9	35.3	39.7	39.3	39.6
Institution’s acronym^d^	10.1	9.0	8.9	7.7	8.3	7.9	7.8
Name of regional/local office^d^	10.1	9.0	8.9	7.7	8.3	7.9	7.8
CNPJ of regional/local office^d^	10.1	9.0	8.9	7.7	8.3	7.9	7.8
Another type of supply^d^	3.2	3.9	4.3	11.1	4.9	5.2	5.3

a) Mandatory field for any form of supply; b) IBGE: Instituto Brasileiro de Geografia e Estatística; c) Mandatory field for at least one form of water supply; d) Non mandatory field; e) SAA: Water Supply System; f) SAC: Alternative Collective Solution.

The results (Tables 1 and 2) show that 14 variables (n = 35) were classified as excellent, as they had 100.0% completeness. These are mandatory variables, with essential information on the registration of forms of water supply.

**Table 2:** Average percentage of initial and final completeness of records of forms water supply, Drinking Water Quality Surveillance Information System (SISAGUA), Brazil, 2014-2020

Variable	Average of initial completeness (%)	Quality	Average of final completeness (%)	Final incompleteness (%)	Quality
Geographical region^a^	100.0	Excellent	100.0	0.0	Excellent
Federative Unit	100.0	Excellent	100.0	0.0	Excellent
Regional Health Care^a^	100.0	Excellent	100.0	0.0	Excellent
Municipality^a^	100.0	Excellent	100.0	0.0	Excellent
IBGE Code^a,b^	100.0	Excellent	100.0	0.0	Excellent
Type of form of supply^a^	100.0	Excellent	100.0	0.0	Excellent
Form of water supply code^a^	100.0	Excellent	100.0	0.0	Excellent
Name of the form of water supply^a^	100.0	Excellent	100.0	0.0	Excellent
Reference year^a^	100.0	Excellent	100.0	0.0	Excellent
Date of registration^a^	100.0	Excellent	100.0	0.0	Excellent
Date of completion^a^	100.0	Excellent	100.0	0.0	Excellent
Surface water collection^a^	100.0	Excellent	100.0	0.0	Excellent
Underground water collection^a^	100.0	Excellent	100.0	0.0	Excellent
Inhabitant/household ratio^a^	100.0	Excellent	100.0	0.0	Excellent
Number of residential economies (permanent households)^a^	100.0	Excellent	99.9	0.1	Excellent
Filtration^c^	95.2	Excellent	91.1	8.9	Good
Disinfection^c^	94.7	Good	90.2	9.8	Good
Cistern^d^	88.4	Regular	100.0	0.0	Excellent
Rainwater harvesting^d^	88.4	Regular	100.0	0.0	Excellent
Water tank^d^	47.4	Very poor	100.0	0.0	Excellent
No reservoir^d^	47.4	Very poor	100.0	0.0	Excellent
SAA/SAC provide water to the population^c,e,f^	47.4	Very poor	100.0	0.0	Excellent
Water tank truck^d^	41.0	Very poor	100.0	0.0	Excellent
Fountain^d^	41.0	Very poor	100.0	0.0	Excellent
Spring^d^	41.0	Very poor	100.0	0.0	Excellent
Pipeline^d^	41.0	Very poor	100.0	0.0	Excellent
SAAc,^e^ provides water to the population	41.0	Very poor	100.0	0.0	Excellent
Number of residential economies (occasional use)^d^	39.4	Very poor	52.5	47.5	Poor
Institution type^d^	38.7	Very poor	73.8	26.2	Regular
Institution name^d^	38.7	Very poor	73.8	26.2	Regular
CNPJ of the institution^d^	38.7	Very poor	73.8	26.2	Regular
Institution’s acronym^d^	8.5	Very poor	15.9	84.1	Very poor
Name of regional/local office^d^	8.5	Very poor	15.9	84.1	Very poor
CNPJ of regional/local office^d^	8.5	Very poor	15.9	84.1	Very poor
Another type of supply^d^	5.4	Very poor	6.4	93.6	Very poor

a) Mandatory field for any form of supply; b) IBGE: Instituto Brasileiro de Geografia e Estatística; c) Mandatory field for at least one form of water supply; d) Non mandatory field; e) SAA: Water Supply System; f) SAC: Alternative Collective Solution.

The variable 'number of residential economies (permanent households)' showed 402 (0.1%) records with an empty field, being classified as excellent. For the variable 'filtration', 40,306 (8.9%) empty records were identified, and for the variable 'disinfection' 44,061 (9.8%), therefore, their level of completeness was classified as good.

'Cistern' and 'rainwater harvesting' are variables only for SAC and SAI. The variables 'water tank', 'no reservoir' and 'SAA/SAC provide water to the population' are variables present only in SAI. The variables 'water tank truck', 'fountain', 'spring', 'pipeline' and 'SAA provides water to the population' are exclusive to SAC. After filtering these variables, according to their filling rules, excellent completeness (100.0%) was verified.

The variable 'number of residential economies (occasional use)', that was present only for SAA and SAC, had 105,354 unfilled records, 47.5% incompleteness. The variables 'institution type', 'institution name' and 'CNPJ (National Registry of Legal Entities) of the institution' did not contain records for the type of SAI and showed 26.2% data incompleteness (118,279 records) each, thus, their completeness was classified as regular.

The variables 'institution’s acronym', 'name of regional/local office' and 'CNPJ of regional/local office', which are used only for state companies and they are not present in SAI, had 378,976 (84.1%) unfilled records, constituting a very poor completeness. For the variable 'another type of supply', the classification was also very poor, with 715,403 (93.6%) empty records.

Of the total of 35 variables, 15 had their classification adjusted after considerations of SISAGUA’s filling rules; and of these, ten variables were reclassified as excellent after the appropriate considerations about the filling rules.

## Discussion

Taking into account SISAGUA’s operational rules, the system showed an excellent classification for 25 variables, good for two, regular for three, poor for one and very poor for four variables. For most variables, the system showed excellent data completeness. Similar to the evaluation of the completeness of information systems on public health budgets,^11^ this study addressed a dimension of health surveillance that has not been explored yet. Several studies have checked the completeness of epidemiological databases.^11^ ^-^ ^19^ However, we could not find any studies that had evaluated this attribute for SISAGUA data.

The variable 'number of residential economies (permanent households)' showed unfilled records, even though it was a mandatory variable, and this situation was present in all the years of the study period. Possibly, this is a persistent failure, difficult to identify the problem and implement a definitive resolution. However, the number of inconsistent records was low and this variable maintained its classification as excellent; a fact that was also observed in the evaluation of the completeness of dengue notifications (2007-2015) in Fundão/ES, where filling in below 100% was identified in mandatory fields.^19^

The adoption of corrective measures for inconsistent data in health information systems is essential in order to improve the credibility of information, improving the veracity of indicators and contributing to optimize public health action plans.^20^

The records left blank for the variables 'filtration' and 'disinfection' resulting from Boolean questions (yes; no), indicate their existence or not in the water treatment process. However, this is an optional field for SAI, that is, when one of the options is not selected, the field is not filled in and remains empty (in blank).

The variable 'number of residential economies (occasional use)' showed very poor completeness, a result possibly related to the fact that it is an optional field, besides many forms of water supply do not present value for this variable. Some variables related to the institutions responsible for water supply had poor or very poor completeness results, which may be related to the fact that, for SAC, there is not always an institution responsible for the form of supply.

The variable 'another type of supply' showed the worst percentage of completeness. This variable is part of a set of information related to the type of water supply provided by SAC or SAI, and it is an open and non-mandatory field.

As verified in this study, other studies, such as an evaluation of tuberculosis records on the Notifiable Health Conditions Information System (SINAN) in Santa Catarina (2007-2016),^11^ and another one on notifications of violence perpetrated against children on the Violence and Accident Surveillance System for Urgency and Emergency Sentinel Services (VIVA) in Pernambuco (2009-2012),^14^ pointed out that, despite a significant number of mandatory variables, which corroborates an excellent data completeness, the optional variables present a high rate of incompleteness in the database. This finding makes it necessary to adopt measures to improve this result. Therefore, it is worth considering the mandatory filling out of fields, as well as investments in raising awareness of the importance of filling in fields completely and the relevance of information produced using these data.

Good quality of existing data in health information systems is crucial for planning, decision making and monitoring of health actions. The Ministry of Health makes permanent investments to ensure its operationalization,^12^ ^-^ ^15^ and all this effort and investment made are lost when the correct information do not enter onto the systems.^19^ With regard to SISAGUA, the absence of information compromises the characterization of the water supply in the country.

This study presents as limitations, several versions of the variable structure, making it difficult to build historical series. Moreover, it is a fairly recent system, with little scientific production on the subject, which makes comparisons difficult. SISAGUA has a particular logical construction, unlike other systems because it is not directed to a health condition, which can still cause difficulties to the traditional epidemiological model.

Taking these results, it can be concluded that SISAGUA has excellent data completeness, although it reveals areas for improvement. Since it is a complex system, it is necessary to know how it works and its rules, aiming at a reliable analysis and interpretation of data. This type of study contributes to a continuous improvement of SISAGUA and enables the identification of inconsistencies and weaknesses in the quality of its data.
